# Covariation of the amplitude and latency of motor evoked potentials elicited by transcranial magnetic stimulation in a resting hand muscle

**DOI:** 10.1007/s00221-023-06575-z

**Published:** 2023-02-22

**Authors:** A. M. Vallence, B. K. Rurak, H. Fujiyama, G. R. Hammond

**Affiliations:** 1 School of Psychology, College of Health and Education, Murdoch University, 90 South Street, Murdoch, WA 6150 Australia; 2 Centre for Healthy Ageing, Health Futures Institute, Murdoch University, Murdoch, WA 6150 Australia; 3 Centre for Molecular Medicine and Innovative Therapeutics, Murdoch, WA 6150 Australia; 4 School of Psychological Science, Faculty of Science, University of Western Australia, Perth, WA Australia

**Keywords:** Motor cortex, Transcranial magnetic stimulation, Motor evoked potential amplitude, Motor evoked potential latency, Variability, Movement disorders

## Abstract

Transcranial magnetic stimulation (TMS) is a non-invasive brain stimulation technique used to study human neurophysiology. A single TMS pulse delivered to the primary motor cortex can elicit a motor evoked potential (MEP) in a target muscle. MEP amplitude is a measure of corticospinal excitability and MEP latency is a measure of the time taken for intracortical processing, corticofugal conduction, spinal processing, and neuromuscular transmission. Although MEP amplitude is known to vary across trials with constant stimulus intensity, little is known about MEP latency variation. To investigate MEP amplitude and latency variation at the individual level, we scored single-pulse MEP amplitude and latency in a resting hand muscle from two datasets. MEP latency varied from trial to trial in individual participants with a median range of 3.9 ms. Shorter MEP latencies were associated with larger MEP amplitudes for most individuals (median *r* = − 0.47), showing that latency and amplitude are jointly determined by the excitability of the corticospinal system when TMS is delivered. TMS delivered during heightened excitability could discharge a greater number of cortico-cortical and corticospinal cells, increasing the amplitude and, by recurrent activation of corticospinal cells, the number of descending indirect waves. An increase in the amplitude and number of indirect waves would progressively recruit larger spinal motor neurons with large-diameter fast-conducting fibers, which would shorten MEP onset latency and increase MEP amplitude. In addition to MEP amplitude variability, understanding MEP latency variability is important given that these parameters are used to help characterize pathophysiology of movement disorders.

## Introduction

Transcranial magnetic stimulation (TMS) is a safe, non-invasive brain stimulation technique that has been used frequently over the past three decades to study human neurophysiology (Barker et al. 1985; Hallett 2007). A single, suprathreshold TMS pulse delivered to the primary motor cortex (M1) elicits a motor evoked potential (MEP) in the muscle(s) controlled by the cortical representation(s) over which the pulse was delivered. The MEP is the net result of a complex descending corticospinal volley that can comprise a series of components with a direct (D) wave from stimulation of the axons of corticospinal cells and indirect (I) waves from activation of corticospinal cells by interneurons within M1. MEP amplitude is a measure of corticospinal excitability (Hallett 2007) and MEP latency is a measure of the sum of the time taken for intracortical processing, corticofugal conduction, and neuromuscular transmission.

It is well known that MEP amplitude is variable from trial to trial when tested under the same conditions. Recent work has shown a minimum of 20 single-pulse TMS trials is required for a reliable mean MEP amplitude (Goldsworthy et al. 2016; Biabani et al. 2018). Research focused on understanding the factors that influence MEP variability suggests a role of oscillations in neural excitability, recorded as rhythmic electrical activity generated by populations of neurons. Alpha and beta oscillations have been reported to have opposing phase-dependent influences on MEP amplitude, with larger MEPs when TMS is applied at the trough than the peak of alpha oscillations (Bergmann et al. 2019; Desideri et al. 2019) and when TMS is applied at the peak than the trough of beta oscillations (Khademi et al. 2019; Torrecillos et al. 2020). Furthermore, MEP amplitude fluctuations have been shown to follow much slower periodicities, with larger MEPs when TMS is applied at that peak than the trough of slow cortical oscillations (< 1 Hz) (Bergmann et al. 2012). Although MEP latency variability has been studied much less than MEP amplitude variability, one report suggests that these two MEP characteristics might covary: both MEP amplitude and latency vary with the phase of beta oscillations and slow oscillations, with shorter latencies (range 0.2–0.9 ms) and larger amplitudes at the peak than the trough (Bergmann et al. 2012; Torrecillos et al. 2020).

We scored the amplitude and latency of MEPs evoked by single TMS pulses in the resting first dorsal interosseous (FDI) from two experimental datasets gathered by different researchers following different experimental protocols. Experiment 1 measured short-interval intracortical inhibition (SICI) at two inter-stimulus intervals (ISIs), the ISI that produced maximal short-interval intracortical facilitation (SICF) and at the ISI that produced minimal SICF (data from this experiment have not been published). Experiment 2 assessed the test–retest reliability of SICF in two separate experimental sessions (Qasem et al. 2020). The data from both experiments show large variability in MEP amplitude and latency, and a clear negative association of latency and amplitude of MEPs evoked by single pulses within a single session (Experiment 1) and different sessions (Experiment 2). The primary aim of the current study was to examine onset latency variability for MEPs elicited by suprathreshold single-pulse TMS in the resting hand muscle. A secondary, exploratory aim, was to examine the association between onset latency and amplitude for MEPs elicited by suprathreshold single-pulse TMS in the resting hand muscle. In both experiments the negative association between MEP latency and amplitude was evident in individual subjects.

## Methods

### Participants

Data were analyzed from two experiments. Data from 25 adults (13 females; median age 24 years, range: 18–35 years) from Experiment 1 and five adults (5 females; median age 21 years, range: 21–26 years) from Experiment 2 were included in the analysis. Data from an additional 15 participants were collected in Experiment 2 but an artifact from the TMS pulse in the electromyographic (EMG) recording in some trials led to uncertainty in determining MEP onset, and hence there were insufficient trials for the data from these participants to be included in the analysis. Participants were recruited from Murdoch University and the local community. The protocol was performed in accordance with the Declaration of Helsinki and approved by the Murdoch University Human Research Ethics Committee. All participants gave written informed consent prior to testing and were screened for any conditions that would contraindicate TMS (Rossi et al. 2011; Rossini et al. 2015a, b).

### TMS

In both experiments, EMG activity was recorded from the relaxed FDI of the right hand using Ag-AgCI surface electrodes placed in a belly–tendon montage. The EMG signal was amplified (× 1000; CED 1902 amplifier), bandpass filtered (20–1000 Hz) and digitized at a sampling rate of 2 kHz (CED 1401 interface). Two Magstim 200^2^ stimulators connected by a MagStim BiStim^2^ module (Magstim Co., Whitland, Dyfed, UK) were used to generate single and paired monophasic pulses: here we report data only from single-pulse trials. Pulses were delivered through a figure-of-eight coil (90-mm diameter) placed tangentially to the left M1 with the handle positioned backwards and ~ 45º away from the midline to induce posterior–anterior current flow in the cortex. The optimal site, resting motor threshold (RMT), and TMS intensity to elicit MEPs ~ 1 mV in peak-to-peak amplitude (SI_1mV_) were obtained for the relaxed FDI muscle. RMT was defined as the minimum stimulus intensity (as a percentage of maximum stimulator output; MSO) required to elicit MEPs ≥ 50 µV in at least five out of 10 consecutive trials (Rossini et al. 2015a, b). SI_1mV_ was defined as the stimulus intensity (as a % of MSO) required to evoke a MEP with a mean peak-to-peak amplitude of ~ 1 mV.

### Experimental protocol

The original experiment was designed to measure SICI at the ISI that produced maximal SICF (‘SICI at SICF Peak’) and the ISI that produced minimal SICF (‘SICI at SICF Trough’). Eight experimental blocks of single- and paired-pulse TMS trials were delivered: four blocks included trials optimized to measure SICI at SICF Peak and four blocks included trials optimized to measure SICI at SICF Trough (order counterbalanced across participants). Although not the focus of the current manuscript, SICI was measured with a subthreshold conditioning stimulus followed by a suprathreshold test stimulus, and SICF was measured with a suprathreshold test stimulus followed by a subthreshold conditioning stimulus. The inter-stimulus intervals for the paired-pulse trials measuring SICI at SICF Peak and SICI at SICF Trough were ~ 1.5 ms and ~ 2.7 ms, respectively. Each block consisted of 12 single-pulse trials at the intensity of SI_1mV_ and 28 paired-pulse trials (with different conditioning stimulus intensities); trial conditions were pseudo-randomized with an inter-trial interval of 5 s (± 20%). A total of 48 single-pulse trials were delivered to each participant for both the SICI at SICF Peak and SICI at SICF Trough conditions. The amplitude and latency of the MEPs from single-pulse trials are reported here.

*Experiment 2.* To measure the test–retest reliability of SICF across two experimental sessions separated by one week (SICF Session 1 and SICF Session 2), 16 experimental blocks were delivered. Each block consisted of eight single-pulse trials at the intensity of SI_1mV_ and 40 paired-pulse trials with different ISIs; trial conditions were pseudo-randomized with an inter-trial interval of 5 s (± 20%). A total of 128 single-pulse trials were delivered to each participant in both SICF sessions. The amplitude and latency of the MEPs from these single-pulse trials are reported here.

### Data analysis

Trials in which RMS EMG activity exceeded 0.02 mV during the 50 ms prior to TMS were excluded (Opie et al. 2015, Qasem, Fujiyama et al. 2020, Vallence et al. 2021). The peak-to-peak MEP amplitude was scored automatically from 40 ms of EMG activity beginning 10 ms after TMS application. MEP latency was scored manually by one of two experienced experimenters using a custom-made script (Signal, CED) that displayed raw and rectified EMG traces for each trial: one vertical cursor was automatically positioned at TMS onset and a second vertical cursor was manually positioned at the MEP onset by the experimenter; the time (in ms) between the two vertical cursors was scored as the MEP latency. For trials in which either experimenter was uncertain about the MEP onset both experimenters viewed the trial together to determine MEP onset. In Experiment 1, the median percentage of trials from each of the 25 participants deemed uncertain and scored by both experimenters together was 7.9% (range 0.0–34.1%). In Experiment 2, the median percentage of trials from each of the 5 participants deemed uncertain and scored by both experimenters together was 7.5% (range 5.4–9.9%) for Session 1 and 10.6% (range 3.3–22.5%) for Session 2. In rare cases, trials were excluded when the two experimenters agreed that the MEP onset could not be determined precisely either because of fluctuations in the background EMG activity or because the MEP was too small to identify the onset (i.e., the peaks of the MEP did not clearly exceed the background EMG activity). For Experiment 1, the median percentage of trials excluded was 2.2% (range 0.0–52.3%). For Experiment 2, the median percentage of trials excluded was 3.8% (range 1.6–5.9%) for Session 1 and 1.3% (range 0–3.9%) for Session 2.

From Experiment 1, the median number of single-pulse trials included from the dataset that measured SICI at SICF Peak was 43 (range 30–48) and the median number of single-pulse trials included from the dataset that measured SICI at SICF Trough was 43 (range 21–47). From Experiment 2, the median number of single-pulse trials included from the dataset that measured SICF in Session 1 was 126 (range 102–128) and the median number of single-pulse trials included from the dataset that measured SICF in session 2 was 101 (range 79–127). MEP amplitudes and latencies were standardized for each participant with a *z*-score transformation. Associations between MEP amplitude and latency (quantified by Pearson’s *r*) were examined for each individual participant for (1) SICI at SICF Peak, (2) SICI at SICF Trough, (3) SICF Session 1, and (4) SICF Session 2. All values are expressed as mean and standard deviation (SD) unless otherwise specified.

## Results

### Experiment 1: within-session MEP amplitude and latency

**Fig. 1:**
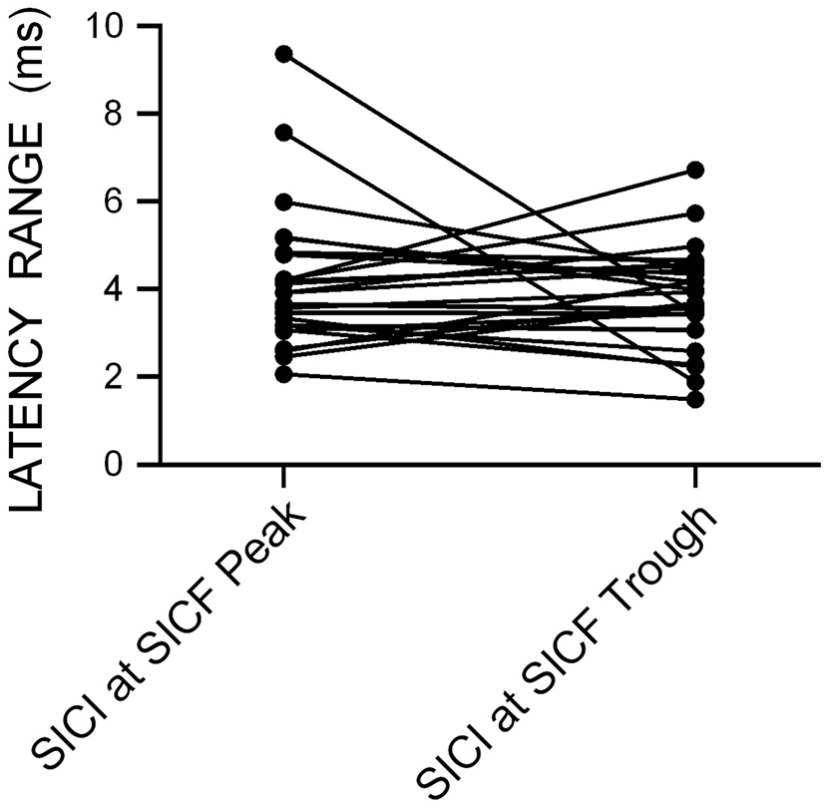
The range of single-pulse TMS MEP latencies (in ms) for each individual from the dataset measuring SICI at SICF Peak and the dataset measuring SICI at SICF Trough

As expected, mean single-pulse MEP amplitude was similar for the SICI at SICF Peak dataset (1.13 mV ± 0.50) and the SICI at SICF Trough dataset (1.14 mV ± 0.65). Mean single-pulse MEP latency was also similar for the SICI at SICF Peak dataset (23.23 ms ± 1.49) and the SICI at SICF Trough dataset (23.30 ms ± 1.56). Figure 1 shows the latency range (the difference between the maximum and minimum latencies in ms) for each individual from the datasets measuring SICI at SICF Peak and SICI at SICF Trough; the range of single-pulse MEP latencies was similar in both datasets (SICI at SICF Peak 4.15 ± 1.62 ms; SICI at SICF Trough 3.82 ± 1.18 ms).

**Fig. 2:**
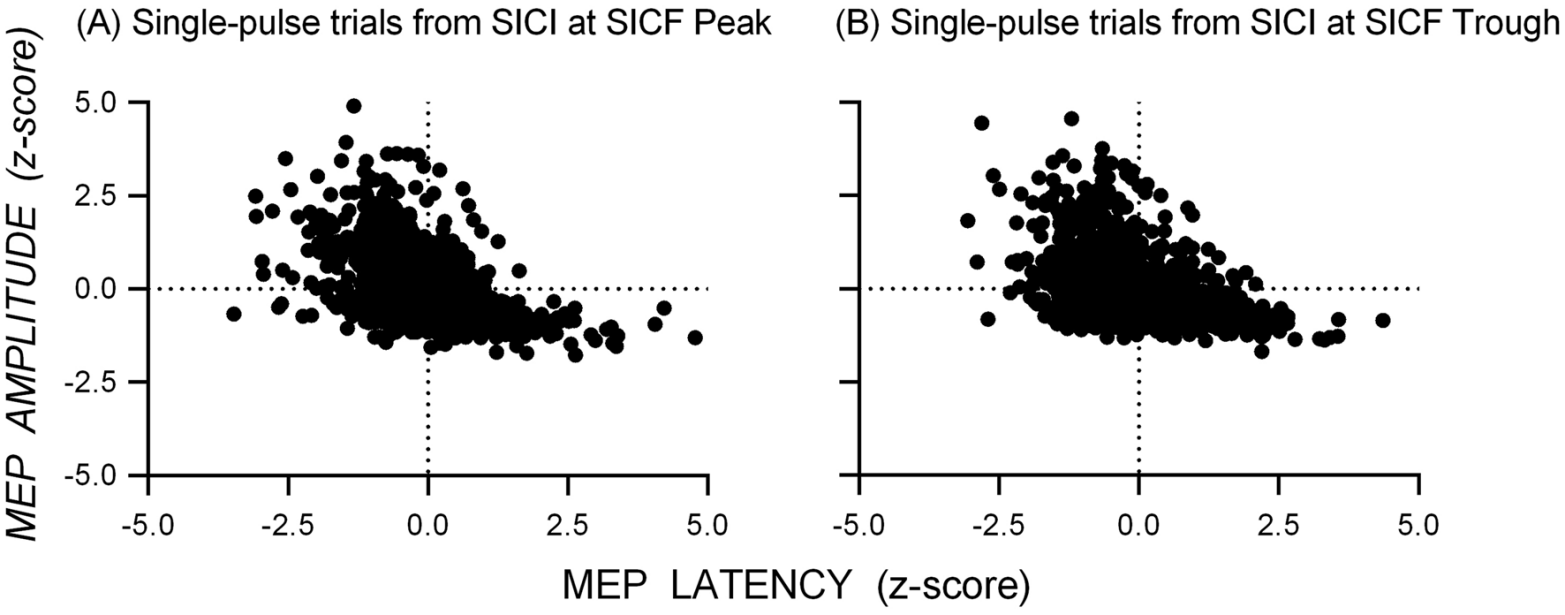
Scatterplots of the standardized MEP amplitude and latency (z-scores) from single-pulse TMS from the dataset measuring SICI at SICF Peak **A** and the dataset measuring SICI at SICF Trough (**B**. Each point shows the latency and amplitude of the MEP from a single trial for each individual participant. For SICI at SICF Peak, the total number of participants was 25 and the total number of trials was 1039; For SICI at SICF Trough, the total number of participants was 25 and the total number of trials was 1036

**Fig. 3:**
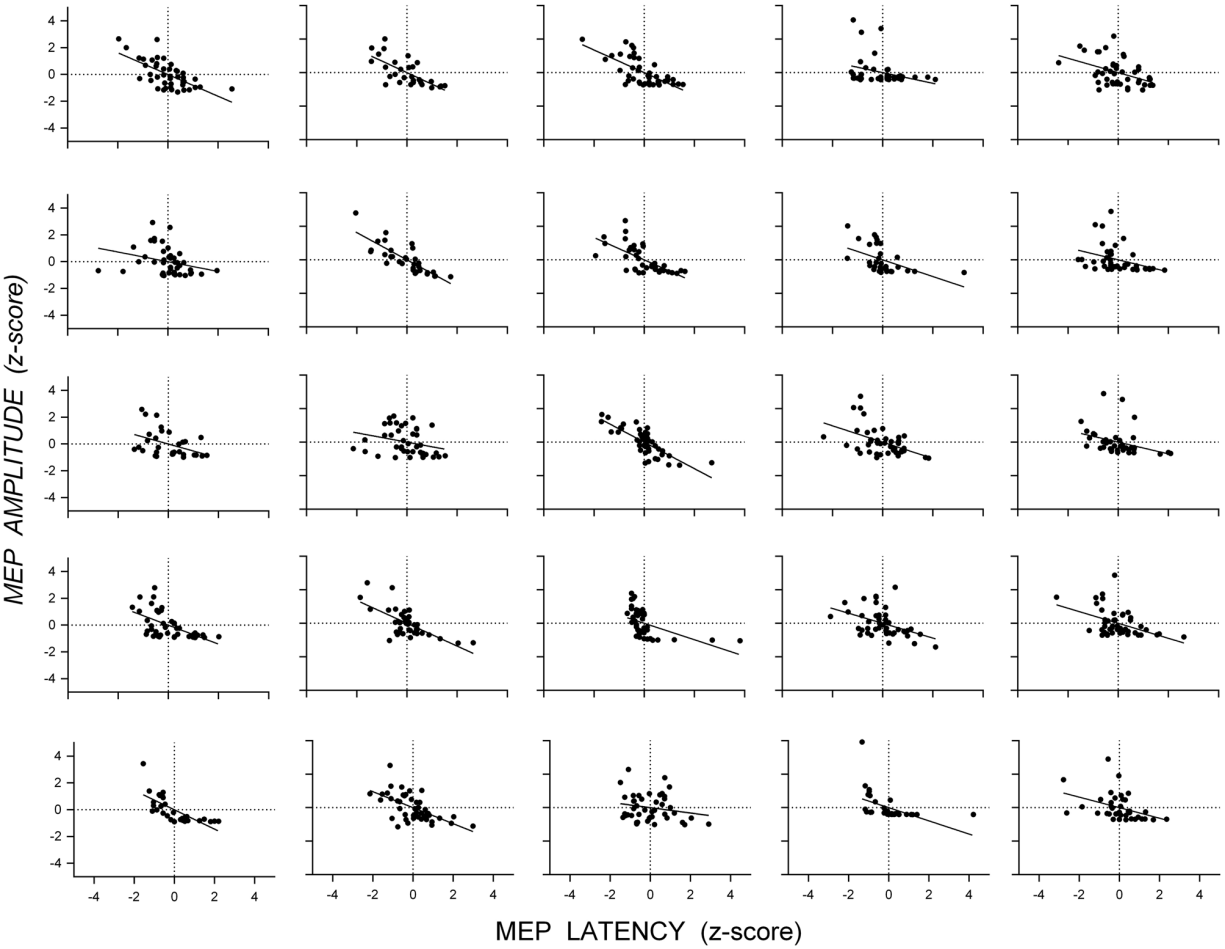
Scatterplots of the standardized MEP amplitude and latency (z-scores) from single-pulse TMS trials for each individual from the dataset measuring SICI at SICF Peak. Each panel shows data from one participant and each point the standardized amplitude and latency of the MEP from each trial

**Fig. 4:**
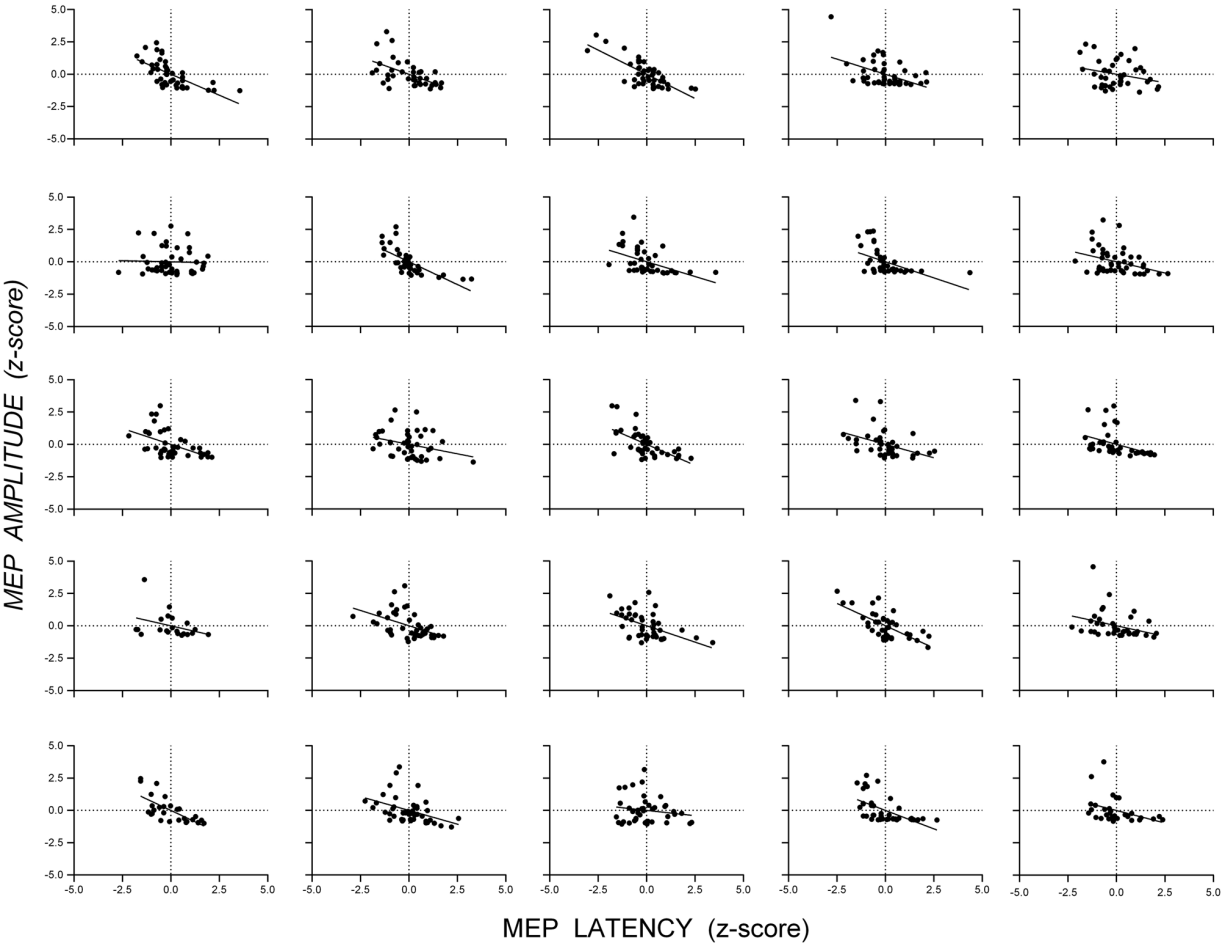
Scatterplots of the standardized MEP amplitude and latency (z-scores) from single-pulse TMS trials for each individual from the dataset measuring SICI at SICF Trough. Each panel shows data from one participant and each point the standardized amplitude and latency of the MEP from each trial

Figure 2 shows scatterplots of the standardized MEP amplitudes and latencies (z-scores) from single-pulse trials for the datasets measuring SICI at SICF Peak (Fig. 2A) and SICI at SICF Trough (Fig. 2B): there was a negative association between MEP amplitude and latency for single-pulse trials in both datasets. The appearance from these data that latency varies continuously with amplitude might be an artifact of aggregating individual data, which could obscure discontinuous changes in latency present in individuals; that is, individuals might show latency clusters associated with different MEP amplitudes. However, scatterplots of the standardized MEP amplitude and latency for each individual from the dataset measuring SICI at SICF Peak (Fig. 3) and SICI at SICF Trough (Fig. 4) reveal that 22 out of 26 individuals showed a negative association between amplitude and latency and that the association was present with continuous changes in both amplitude and latency (SICI at SICF Peak: median *r* = − 0.49, range − 0.20 to − 0.81; SICI at SICF Trough: median *r* = − 0.47, range − 0.03 to − 0.75). It is also clear from Figs. 3 and 4 that there was a ‘basement’ effect present in some individuals, such that latencies of the smallest scoreable MEPs did not continue to increase, attenuating the linear correlation.

### Experiment 2: between-session MEP amplitude and latency

**Fig. 5:**
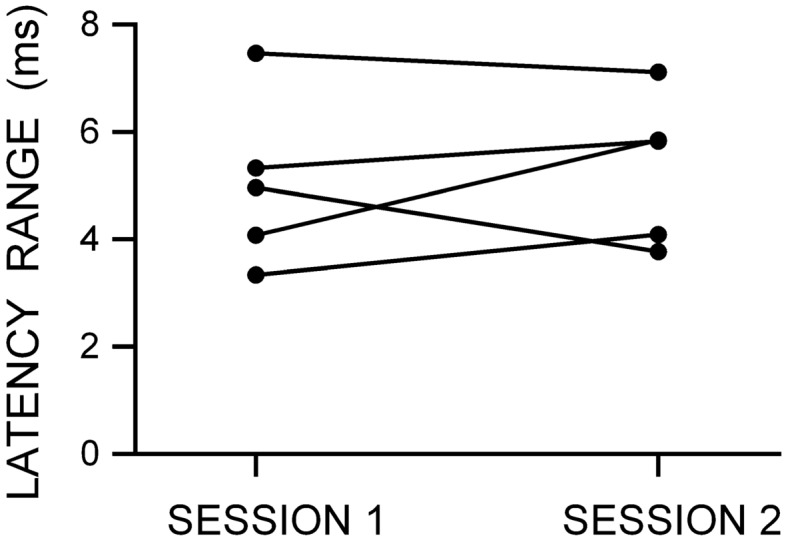
The range of MEP latencies (in ms) for each individual from the dataset measuring SICF Session 1 and SICF Session 2

As expected, single-pulse MEP amplitude was similar for the dataset measuring SICF in Session 1 (1.10 mV ± 0.29) and the dataset measuring SICF in Session 2 (1.11 mV ± 0.59). Mean single-pulse MEP latency was also similar for the SICF Session 1 dataset (22.03 ms ± 1.45) and the SICF Session 2 dataset (21.54 ms ± 1.47). Figure 5 shows the range of MEP latencies (in ms) for each individual from the dataset measuring SICF in Session 1 and Session 2. It is clear from Fig. 5 that the range of single-pulse MEP amplitude and single-pulse MEP latency is similar in both sessions, and similar to the SICI at SICF Peak and SICI at SICF Trough datasets (see Fig. 1).

**Fig. 6:**
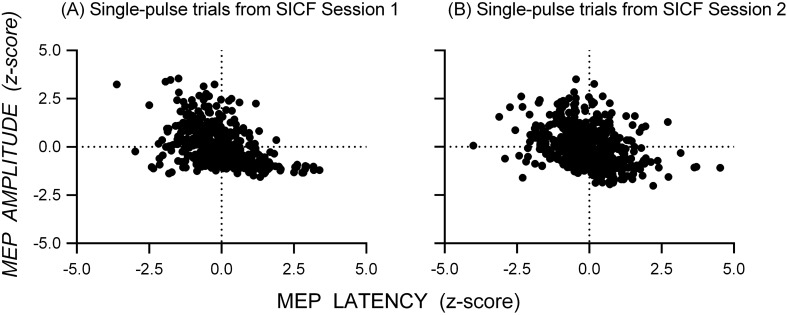
Scatterplots of the standardized MEP amplitude and latency (z-scores) from single-pulse TMS from the dataset measuring SICF in Session 1 **A** and the dataset measuring SICF in Session 2 **B**. Each point shows the latency and amplitude of the MEP from a single trial for each individual participant. The total number of participants was 5 and the total number of trials was 600 for Session 1 and 530 for Session 2

**Fig. 7:**
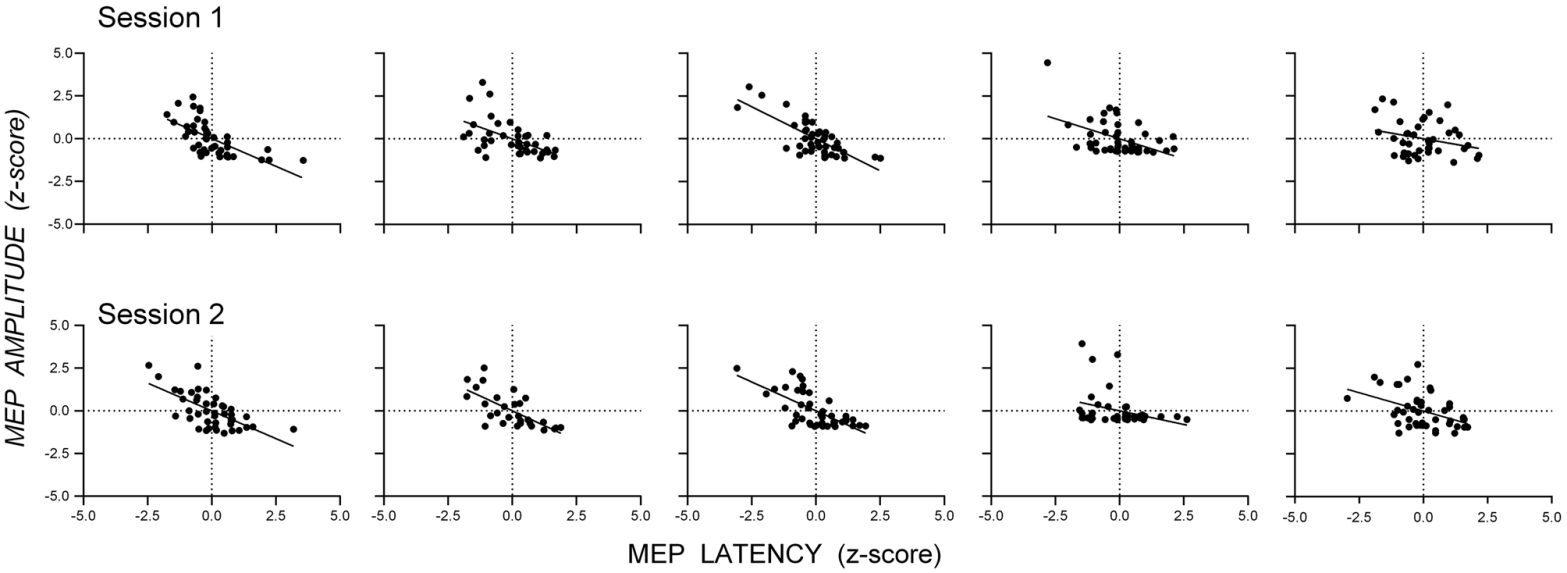
Scatterplots of the standardized MEP amplitude and latency (z-scores) from single-pulse TMS trials for each individual from the dataset measuring SICI at SICF Trough. Each panel shows data from one participant and each point the standardized amplitude and latency of the MEP from each trial

Figure 6 shows scatterplots of the standardized MEP amplitude and latency (z-scores) from single-pulse trials for the datasets measuring SICF in Session 1 (Fig. 6A) and Session 2 (Fig. 6B): there is a strong negative association between MEP amplitude and latency for single-pulse trials in both datasets. Scatterplots of the standardized MEP amplitude and latency for each individual from the dataset measuring SICF in Session 1 and Session 2 (Fig. 7) reveal that the majority of individuals showed a negative association between amplitude and latency and that the association was present with continuous changes in both amplitude and latency (Session 1: median *r* = − 0.45, range − 0.15 to − 0.54; Session 2: median *r* = − 0.45, range − 0.37 to − 0.56).

## Discussion

Although it is well known that single-pulse MEP amplitude varies from trial to trial, little is known about variation in MEP latency. Here, we measured single-pulse MEP amplitudes and latencies from two experimental data sets gathered by different researchers following different experimental protocols with participants at rest and without any task to perform. There are two key findings from the current study. First, individual MEP latencies vary considerably (with a range of ~ 4 ms) from trial to trial. Second, this variation in MEP latency is continuous and is accompanied by a systematic covariation in MEP amplitude, with shorter latencies associated with larger MEP amplitudes. This covariation of MEP amplitude and latency was observed in individual participants both within a single experimental session (Experiment 1) and in different experimental sessions (Experiment 2).

MEP latency is typically estimated by an automatic method that defines latency as the time at which the post-pulse EMG activity exceeds a predetermined threshold level, commonly two standard deviations or 5% above the EMG activity in the 50 ms preceding the pulse (Huang and Mouraux 2015; Hordacre et al. 2017; İşcan et al. 2018; Torrecillos et al. 2020). Previous research has reported intra-class correlation coefficients of 0.75—0.97 for MEP onset latency between different blocks of single-pulse MEPs within experimental sessions (Bastani and Jaberzadeh 2012) and 0.75–0.80 for MEP onset latency between experimental sessions on separate days, indicating excellent test–retest reliability for this measure (Livingston and Ingersoll 2008; Bastani and Jaberzadeh 2012). In the current study, MEP latencies were scored by two experienced researchers positioning a marker at the onset of a visual representation of each MEP, indicated by a sustained deflection of the EMG trace from background EMG activity that continued to form the initial peak of the MEP. Trials on which the onset of the MEP could not be identified conclusively were not considered in the analysis. Although manual scoring of the visual representation of the EMG trace is time-consuming and necessarily subject to some error, it is likely to be more accurate and precise than automatic estimation which might be subject to systematic misidentification of MEP onset, particularly given some evidence that the initial slope of the MEP might vary with its amplitude (İşcan et al. 2018).

The first key finding of the current study is that individuals showed a considerable range of MEP latency from trial to trial, with an overall median MEP latency range of 3.9 ms. The latency ranges observed here show that the time taken by intracortical processing, corticofugal conduction, spinal processing, and neuromuscular transmission (Day et al. 1989) can vary by ~ 4 ms, depending on the state of the corticospinal system when TMS is delivered. The state changes at any point in the corticospinal pathway could influence MEP latency; however, it is likely that the time taken for corticofugal conduction and neuromuscular transmission vary little, if at all, and so is unlikely to have contributed to the observed variation in latency (Hess et al. 1987). Although TMS delivered with a coil orientation inducing posterior-to-anterior current flow is unlikely to elicit a D-wave at low (near threshold) intensities, it is possible that a D-wave could be elicited on some trials in some individuals with the SI_1mV_ intensity (Di Lazzaro et al. 1998). However, the observation that the latency variation in individuals was continuous shows that the time taken by the neural processes that determine latency themselves vary continuously, and not discontinuously, as would be expected if a D-wave, which is known to occur ~ 1.5 ms before I1 (Ziemann and Rothwell 2000; Di Lazzaro et al. 2012), was present in some but not other trials. Therefore, it is possible that some but not all of the MEP onset latency variability could be explained by the presence or absence of a D-wave across trials. The second key finding of the current study is that the continuous variation in MEP latency was accompanied by continuous covariation in peak-to-peak MEP amplitude, with shorter latencies associated with larger amplitudes. This covariation, which was evident in individuals, indicates that latency and amplitude are jointly determined by the excitability of the corticospinal system when TMS is applied. A similar MEP latency range (~ 4 ms) and covariation of MEP amplitude and MEP onset latency has been reported previously following transcranial electrical stimulation of M1 (Day et al. 1987). It has been previously reported that mean MEP onset latency shortens and MEP amplitude increases during voluntary contraction compared to rest (van den Bos et al. 2017); mean MEP onset latency at the group level was 4.2 ms shorter during contraction than rest. In addition, during a voluntary muscle contraction, mean MEP onset latency shortened with increasing stimulus intensity (van der Kamp et al. 1996; Säisänen et al. 2008). None of these studies, however, reported MEP latency range or associations between MEP onset latency and MEP amplitude at the individual trial level. Future research should examine the effect of voluntary contraction and stimulus intensity on both MEP onset latency variability and the covariation of MEP onset latency and MEP amplitude.

The nature of the fluctuations in M1 excitability is not fully understood. In both experiments reported here, many observations were obtained for each individual by applying TMS with inter-trial intervals that varied randomly between 4.5 and 5.5 s without reference to ongoing EEG activity. With this approach, the observed trial-to-trial variation in latency and amplitude of the MEPs will likely reflect moment-to-moment variation in the state of the corticospinal system in each individual (Bergmann et al. 2012; Torrecillos et al. 2020). Previous reports have shown shorter MEP latency and larger MEP amplitude at the peak than the trough of beta oscillations (Torrecillos et al. 2020) and neocortical slow oscillations (< 1 Hz) (Siebner et al. 2009; Bergmann et al. 2012). However, there are reports showing opposing phase-dependent effects with alpha oscillations, specifically, with MEP amplitude greater at the trough than the peak of alpha oscillations (Bergmann et al. 2019; Desideri et al. 2019). Together, this evidence suggests that periodic fluctuations in neural activity at different frequencies indicate changes in cortical excitability (Buzsaki and Draguhn 2004; Buzsáki et al. 2012) and, thus, influence MEP amplitude and latency; however, the exact role of oscillations, and the combination of oscillations at varying frequencies on the covariation of MEP amplitude and latency (with observed range of ~ 4 ms) remains unknown.

Excitability-related variability in MEP latency and amplitude most likely arises from variability of neuronal activity in both the cortex and the spinal cord. At the cortical level, the latency variability seen here with posterior-to-anterior current flow could result from variability in the excitability of the excitatory interneurons in M1 that make monosynaptic connections with the corticospinal cells, and which are responsible for generating I-waves in the descending volley, and of the corticospinal cells themselves (Di Lazzaro et al. 2012; Ziemann 2020). Little is known about I-wave onset latency variability: one non-human primate study delivering direct electrical stimulation to the motor cortex and recording descending volleys from the cervical spinal cord showed that I-wave onset latency decreased by 0.2–0.5 ms as stimulus intensity increased, and that the onset latencies of later I-waves (i.e., I2 and I3) were more variable than those of I1 waves (Kernell and Chien-Ping 1967). It is possible that TMS delivered during a heightened state of excitability, with a subliminal fringe of partially depolarised excitatory interneurons, could shorten I-wave latency by discharging these cells more rapidly than when in a lower state of excitability. Similarly, the target corticospinal cells would be discharged more rapidly if TMS was delivered while some cells were partially depolarised during a state of heightened excitability. TMS delivered during a heightened state of excitability would also discharge a greater number of corticospinal cells, increasing the amplitude and, by recurrent activation of corticospinal cells, the number of descending I-waves. At the spinal level, this increase in the amplitude and number of I-waves would progressively recruit larger spinal motor neurons with large-diameter fast-conducting fibers, which would shorten MEP onset latency and increase MEP amplitude. Together, both cortical and spinal processes could account for the variation seen in MEP onset latency and the covariation of latency and amplitude.

Different coil orientations have been shown to affect MEP onset latency as well as the composition of the descending volley. MEP onset latency is shortest with a lateral–medial (LM) current flow, and shorter with posterior–anterior (PA) current flow than anterior–posterior (AP) current flow (Werhahn et al. 1994; Hordacre et al. 2017). In terms of the descending volley, LM current flow preferentially elicits the D-wave, whereas PA and AP current flow preferentially elicit early and late I-waves, respectively (Sakai et al. 1997; Di Lazzaro et al. 2001). Growing evidence suggests that different coil orientations might activate distinct neuronal populations, resulting in different descending volleys (for review see: Opie and Semmler 2021). Based on the current literature, we speculate that MEP onset latency variability would be smallest and the association between MEP onset latency and MEP amplitude weakest with LM current flow, which preferentially elicits D-waves, and greatest with AP current flow, which preferentially elicits late I-waves. Future research is necessary to examine whether different coil orientations affect MEP onset latency variability or the covariation of MEP onset latency and MEP amplitude.

Previous research has shown that the mean MEP onset latency is 0.4 ms shorter for MEPs elicited from the MEP hotspot (i.e., the site that elicited the largest MEPs) than from the site corresponding to the hand knob of M1 using individual MRI scans (Julkunen et al. 2009). Although coil position was not tracked using neuronavigation in the current study, any trial-by-trial variations in coil position that might have occurred with an experienced TMS researcher targeting a single stimulation site would have been much smaller than the variations in coil position for the two distinct sites that were examined in the Julkunen et al. (2009) study. Furthermore, the mean MEP onset latency difference of 0.4 ms found between two distinct stimulation sites (Julkunen et al. 2009) is much smaller than the ~ 4 ms MEP onset latency range shown in the present results. Therefore, it is unlikely that the MEP onset latency variance reported in the current study resulted from trial-to-trial variations in coil position. Future research, however, would benefit from trial-to-trial neuronavigation tracking of coil position, which would allow exclusion of trials in which the coil position deviated from the target position.

The current findings show that, even when all muscles are at rest, in a stable posture with the participant not engaged in a task, moment-to-moment fluctuations in excitability lead to a large range of MEP latencies that covary with MEP amplitude. Future research investigating factors that contribute to the variability of MEP onset latency and amplitude is important given that these parameters have been used to help characterize pathophysiology of movement disorders since the first use of TMS in humans (Rothwell et al. 1991). Cortical and spinal processes underpinning MEP onset latency and MEP amplitude could explain the abnormalities in these parameters observed in populations with movement disorders, such as Parkinson’s disease (Rossini and Rossi 2007). In addition, given the assumption that these MEP parameters are related to motor performance (Groppa et al. 2012), understanding mechanisms underlying the covariation of MEP onset latency and amplitude could provide important insights into motor performance under a range of conditions, such as central fatigue and healthy aging.

## Data Availability

The data that support the findings of this study are available from the corresponding author upon reasonable request.

## References

[CR1] Barker AT, Jalinous R, Freeston IL (1985). Non-invasive magnetic stimulation of human motor cortex. Lancet.

[CR2] Bastani A, Jaberzadeh S (2012). A higher number of tms-elicited mep from a combined hotspot improves intra- and inter-session reliability of the upper limb muscles in healthy individuals. PLoS ONE.

[CR3] Bergmann TO, Mölle M, Schmidt MA, Lindner C, Marshall L, Born J, Siebner HR (2012). EEG-guided transcranial magnetic stimulation reveals rapid shifts in motor cortical excitability during the human sleep slow oscillation. J Neurosci.

[CR4] Bergmann TO, Lie A, Zrenner C, Ziemann U (2019). Pulsed facilitation of corticospinal excitability by the sensorimotor mu-alpha rhythm. J Neurosci.

[CR5] Biabani M, Farrell M, Zoghi M, Egan G, Jaberzadeh S (2018). The minimal number of TMS trials required for the reliable assessment of corticospinal excitability, short interval intracortical inhibition, and intracortical facilitation. Neurosci Lett.

[CR6] Buzsáki G, Anastassiou CA, Koch C (2012). The origin of extracellular fields and currents — EEG, ECoG, LFP and spikes. Nat Rev Neurosci.

[CR7] Buzsaki G, Draguhn A (2004). Neuronal oscillations in cortical networks. Science.

[CR8] Day BL, Rothwell JC, Thompson PD, Dick JP, Cowan JM, Berardelli A, Marsden CD (1987). Motor cortex stimulation in intact man. 2. Multiple descending volleys. Brain.

[CR9] Day BL, Dressler D, Maertens de Noordhout A, Marsden CD, Nakashima K, Rothwell JC, Thompson PD (1989). Electric and magnetic stimulation of human motor cortex: Surface EMG and single motor unit responses. J Physiol.

[CR10] Desideri D, Zrenner C, Ziemann U, Belardinelli P (2019). Phase of sensorimotor mu-oscillation modulates cortical responses to transcranial magnetic stimulation of the human motor cortex. J Physiol-London.

[CR11] Di Lazzaro V, Oliviero A, Profice P, Saturno E, Pilato F, Insola A, Mazzone P, Tonali P, Rothwell JC (1998). Comparison of descending volleys evoked by transcranial magnetic and electric stimulation in conscious humans. Electroencephalogr Clin Neurophysiol.

[CR12] Di Lazzaro V, Oliviero A, Saturno E, Pilato F, Insola A, Mazzone P, Profice P, Tonali P, Rothwell JC (2001). The effect on corticospinal volleys of reversing the direction of current induced in the motor cortex by transcranial magnetic stimulation. Exp Brain Res.

[CR13] Di Lazzaro V, Profice P, Ranieri F, Capone F, Dileone M, Oliviero A, Pilato F (2012). I-wave origin and modulation. Brain Stimul.

[CR14] Goldsworthy M, Hordacre B, Ridding M (2016). Minimum number of trials required for within-and between-session reliability of TMS measures of corticospinal excitability. Neuroscience.

[CR15] Groppa S, Oliviero A, Eisen A, Quartarone A, Cohen LG, Mall V, Kaelin-Lang A, Mima T, Rossi S, Thickbroom GW, Rossini PM, Ziemann U, Valls-Sole J, Siebner HR (2012). A practical guide to diagnostic transcranial magnetic stimulation: report of an IFCN committee. Clin Neurophysiol.

[CR16] Hallett M (2007). Transcranial magnetic stimulation: a primer. Neuron.

[CR17] Hess CW, Mills KR, Murray NM (1987). Responses in small hand muscles from magnetic stimulation of the human brain. J Physiol.

[CR18] Hordacre B, Goldsworthy MR, Vallence A-M, Darvishi S, Moezzi B, Hamada M, Rothwell JC, Ridding MC (2017). Variability in neural excitability and plasticity induction in the human cortex: a brain stimulation study. Brain Stimul.

[CR19] Huang G, Mouraux A (2015). MEP latencies predict the neuromodulatory effect of ctbs delivered to the ipsilateral and contralateral sensorimotor cortex. PLoS ONE.

[CR20] İşcan Z, Schurger A, Vernet M, Sitt JD, Valero-Cabré A (2018). Pre-stimulus theta power is correlated with variation of motor evoked potential latency: a single-pulse TMS study. Exp Brain Res.

[CR21] Julkunen P, Säisänen L, Danner N, Niskanen E, Hukkanen T, Mervaala E, Könönen M (2009). Comparison of navigated and non-navigated transcranial magnetic stimulation for motor cortex mapping, motor threshold and motor evoked potentials. Neuroimage.

[CR22] Kernell D, Chien-Ping W (1967). Responses of the pyramidal tract to stimulation of the baboon's motor cortex. J Physiol.

[CR23] Khademi F, Royter V, Gharabaghi A (2019). State-dependent brain stimulation: power or phase?. Brain Stimul.

[CR24] Livingston SC, Ingersoll CD (2008). intra-rater reliability of a transcranial magnetic stimulation technique to obtain motor evoked potentials. Int J Neurosci.

[CR25] Opie GM, Semmler JG (2021). Preferential activation of unique motor cortical networks with transcranial magnetic stimulation: a review of the physiological, functional, and clinical evidence. Neuromodulation.

[CR26] Opie GM, Ridding MC, Semmler JG (2015). Age-related differences in pre-and post-synaptic motor cortex inhibition are task dependent. Brain Stimul.

[CR27] Qasem H, Fujiyama H, Rurak BK, Vallence AM (2020). Good test–retest reliability of a paired-pulse transcranial magnetic stimulation protocol to measure short-interval intracortical facilitation. Exp Brain Res.

[CR28] Rossi S, Hallett M, Rossini PM, Pascual-Leone A (2011). Screening questionnaire before TMS: an update. Clin Neurophysiol.

[CR29] Rossini PM, Rossi S (2007). Transcranial magnetic stimulation. Neurology.

[CR30] Rossini PM, Burke D, Chen R, Cohen LG, Daskalakis Z, Di Iorio R, Di Lazzaro V, Ferreri F, Fitzgerald PB, George MS, Hallett M, Lefaucheur JP, Langguth B, Matsumoto H, Miniussi C, Nitsche MA, Pascual-Leone A, Paulus W, Rossi S, Rothwell JC, Siebner HR, Ugawa Y, Walsh V, Ziemann U (2015). Non-invasive electrical and magnetic stimulation of the brain, spinal cord, roots and peripheral nerves: Basic principles and procedures for routine clinical and research application. An updated report from an I.F.C.N. Committee. Clin Neurophysiol.

[CR31] Rossini PM, Burke D, Chen R, Cohen LG, Daskalakis Z, Di Iorio R, Di Lazzaro V, Ferreri F, Fitzgerald PB, George MS, Hallett M, Lefaucheur JP, Langguth B, Matsumoto H, Miniussi C, Nitsche MA, Pascual-Leone A, Paulus W, Rossi S, Rothwell JC, Siebner HR, Ugawa Y, Walsh V, Ziemann U (2015). Non-invasive electrical and magnetic stimulation of the brain, spinal cord, roots and peripheral nerves: Basic principles and procedures for routine clinical and research application. An updated report from an IFCN Committee. Clin Neurophysiol.

[CR32] Rothwell JC, Thompson PD, Day BL, Boyd S, Marsden CD (1991). Stimulation of the human motor cortex through the scalp. Exp Physiol.

[CR33] Säisänen L, Pirinen E, Teitti S, Könönen M, Julkunen P, Määttä S, Karhu J (2008). Factors influencing cortical silent period: Optimized stimulus location, intensity and muscle contraction. J Neurosci Methods.

[CR34] Sakai K, Ugawa Y, Terao Y, Hanajima R, Furubayashi T, Kanazawa I (1997). Preferential activation of different i waves by transcranial magnetic stimulation with a figure-of-eight-shaped coil. Exp Brain Res.

[CR35] Siebner HR, Hartwigsen G, Kassuba T, Rothwell JC (2009). How does transcranial magnetic stimulation modify neuronal activity in the brain? Implications for studies of cognition. Cortex.

[CR36] Torrecillos F, Falato E, Pogosyan A, West T, Di Lazzaro V, Brown P (2020). Motor cortex inputs at the optimum phase of beta cortical oscillations undergo more rapid and less variable corticospinal propagation. J Neurosci.

[CR37] Vallence A-M, Dansie K, Goldsworthy MR, McAllister SM, Yang R, Rothwell JC, Ridding MC (2021). Examining motor evoked potential amplitude and short-interval intracortical inhibition on the up-going and down-going phases of a transcranial alternating current stimulation (tacs) imposed alpha oscillation. Eur J Neurosci.

[CR38] van den Bos MA, Geevasinga N, Menon P, Burke D, Kiernan MC, Vucic S (2017). Physiological processes influencing motor-evoked potential duration with voluntary contraction. J Neurophysiol.

[CR39] van der Kamp W, Zwinderman AH, Ferrari MD, van Dijk JG (1996). Cortical excitability and response variability of transcranial magnetic stimulation. J Clin Neurophysiol.

[CR40] Werhahn KJ, Fong JK, Meyer BU, Priori A, Rothwell JC, Day BL, Thompson PD (1994). The effect of magnetic coil orientation on the latency of surface EMG and single motor unit responses in the first dorsal interosseous muscle. Electroencephalogr Clin Neurophysiol.

[CR41] Ziemann U (2020). I-waves in motor cortex revisited. Exp Brain Res.

[CR42] Ziemann U, Rothwell JC (2000). I-waves in motor cortex. J Clin Neurophysiol.

